# What makes helpful online mental health information? Empirical evidence on the effects of information quality and responders’ effort

**DOI:** 10.3389/fpsyg.2022.985413

**Published:** 2022-11-29

**Authors:** Cui Guo, Xinying Guo, Guoxin Wang, Shilin Hu

**Affiliations:** ^1^Public Administration, Law School, Panzhihua University, Panzhihua, China; ^2^Technology Economy and Management, Department of Business Administration, Business School, Shantou University, Shantou, China; ^3^School of Business, Renmin University of China, Beijing, China

**Keywords:** helpfulness, health information, interactive feedback, elaboration likelihood model, online mental health communities

## Abstract

Although online health communities are popular in supporting mental health, factors leading to the helpfulness of mental health information are still under-investigated. Based on the elaboration likelihood model and motivation theory, we incorporate two types of health information-related constructs, i.e., information quality (central route) and responders’ effort (peripheral route), and adopt reputation as an extrinsic motivation to build our model. We crawl data from a Chinese online mental health community and extract 11 key variables, and then analyze the model with negative binomial regression. The empirical results indicate that the effect of the length of health information on its helpfulness votes is positively significant, while the effect of readability of health information on its helpfulness votes is relatively negative. In terms of responders’ effort, both the timelines of the response and interactive feedback have a significant positive impact on helpfulness of health information votes, while these effects are negatively moderated by the online reputation of responders. This study contributes to the literature on information evaluation mechanisms in online health communities.

## Introduction

Powered by the rapid development of emerging information and communication technologies (ICTs) and the enhancement of public health awareness during the COVID-19 pandemic, massive online data and information concerning healthcare are growing at an exponential rate (Liu et al., 2020). For instance, online health communities (OHCs) such as *Patientslikeme* and *Haodf.com*, are virtual community for health and medical information generation and transmission, which allow online communication, emotional support and mutual assistance, with doctors, nurses, patients, and their families as the main service objects (Yan and Tan, 2014; Zhou, 2018). Some online health communities are used to support mental health (online mental health communities, OMHCs) and recently has increasingly become important platforms for mental health information seeking and sharing (Liu et al., 2020; Qianqian Ben et al., 2020; Yan et al., forthcoming; Zhou et al., forthcoming). The participation of patients in mental online health services can promote the information as well as emotional support among those peers (Zhou, 2018), so as to improve their health status and enhance their self-health management (Yan and Tan, 2014; Chen et al., 2019).

Although the popularity of OMHCs provides a convenient channel for patients to access services, the complexity of mental illness and treatment methods, people’s low level of mental health literacy, the inherent expertise of health information and knowledge make the information asymmetry (Yan et al., forthcoming), results in higher requirements for identification of pedestrian users (Diviani et al., 2015). In order to reduce search costs of help-seekers without adequate health literacy, and help them identify high-quality information, some affordance features concerning helpfulness evaluation have been adopted by OHCs, such as voting systems (e.g., “thumb” and “virtual gift”), recommendation systems, and electronic word of mouth (eWOM; Guo et al., 2017). Information helpfulness generally refers to the user’s perception of information utility. Helpfulness votes could help users identify information quality, attenuate individual overload cognitive cost in information searches and adoption, thereby enhancing users’ OMHCs’ engagement and continue using behaviors. Additionally, prior studies have shown that information helpfulness votes by users is also conducive to stimulating continuous knowledge contribution and voluntary recommendations of the information contributors (Chen et al., 2019).

In the existing literature, scholars have explored the influential factors affecting online information helpfulness using various lenses and have mainly utilized two perspectives (Racherla and Friske, 2012): information source (i.e., individual characteristics of the information providers), information quality (i.e., the content of the information) (Chen et al., 2021). The majority of studies based on information sources in online comment communities find that reviewers own factors have a direct positive impact on the perceived helpfulness of comments (Qigeng et al., 2017). Moreover, since professionalism can reduce uncertainty, the degree of professionalism of commentators has had a positive influence on the perceived helpfulness of comments (Qazi et al., 2016). In addition, due to the users’ desire for timely responses, the promptness of the response also affects the helpful evaluation of information. As an overall evaluation of information content, the degree of the influence of information quality has a more persistent impact on information helpfulness through the readers’ decision-making process (Mudambi and Schuff, 2010). Furthermore, the authenticity, consistency, sentiment and readability of information also affect the helpfulness of information. These relationships may also be moderated by variables such as the searcher’s self-efficacy in terms of information acquisition, the responder’ s reputation, and the categories of problems (Hernández-Ortega, 2018). However, the above findings mostly focus on the helpfulness of user generated content (UGC) in normal virtual communities, while more attention should be paid to expand on the existing research. For instance, a great number of studies analyze the helpfulness of comments in transactional virtual communities, such as online shopping platforms, which only focus on the impact of online word-of-mouth and customer consumption behavior such as user comments (Jiang and Benbasat, 2004). For knowledge sharing communities, attention is mostly paid to social Q&A platforms such as “Yahoo Q&A” and “Baidu Zhidao” (John et al., 2011; Salloum et al., 2018). Thus, the underlying factors leading to helpfulness of health information in OHCs, especially those relative to mental healthcare, remain under-investigated.

Accordingly, we formulate two research questions from the two following knowledge gaps. Firstly, the majority of studies explore the helpfulness of information from the perspectives of information content and information sources, and rarely pay attention to the responders’ efforts during the interactions between information help-seekers and help-providers (Racherla and Friske, 2012). In OMHCs, responders’ information sharing is presumably prosocial behavior. Different from physical diagnosis or medicine taking consulting, most non-clinical mental patients are only seeking secret releasing and expecting mental information and emotional support. Yet the interactions between help-seeker and help-providers are not limited to a one-time service, but are continuous and dynamic. Focusing on this basis, we propose that users’ helpfulness perception is derived from two sources: one is health information quality, the intuitively obvious nature of the information; another one is responders’ effort, which refers to extra efforts made by the responders that help-seekers or other users could observe. For example, a continuous and additional explanation beyond a main response, in a form of reviews or comments. However, it is unclear which factor is play dominant role during the evaluation of mental health usefulness. Hence, it is necessary to discuss the effect of such two factors on the helpfulness of information provided to the help-seekers by the responders. Accordingly, we propose the following research question 1(RQ1): *How do information quality and responders’ effort influence the helpfulness votes of health information in OMHCs*?

Secondly, In OMHCs, the efforts made by responders could be seen as prosocial behavior driven by intrinsic motivation like altruism or self-actualization. However, the affordance of online reputation ranking, as one kind of most-adopted extrinsic motivation, can avoid the potential risks brought on by false information and even regulate the voluntary contribution behavior of community users. Most studies aim to explain the relationship between reputation and continuous knowledge sharing behavior, and there is limited literature on the impact of reputation on the knowledge seeker’s helpfulness voting. The rewards of the reputation system act as extrinsic motivation, but only affect the quantity of information shared and not the quality. However, those health professionals or experienced users with a high level of reputations have pressure to keep their positive profiles and choose to share knowledge through answering normal users’ questions (Zhou et al., 2018), which is time-consuming and may reduce their extra efforts invested into daily continues communication. Additionally, although some studies have examined the relationships between intrinsic and extrinsic motivations and how their interaction affects individuals’ prosocial behaviors in online communities, their conclusions are inconsistent (Zhao et al., 2016; Khern-am-nuai et al., 2018; Lee and Suzuki, 2020). Hence, we examine whether the effect of responders’ efforts such as interactive feedback among users to the helpfulness of votes could be strengthen or weaken in OMHCs. we put forward the research question 2 (RQ2): *How do responders’ online reputation affect the relationship between responders’ effort and the helpfulness votes of health information in OMHCs?*

In order to answer the above questions, we take an online mental health service platform in China as example, and construct variables affecting helpfulness of information by collecting Q&A data from the platform for 3 months. Based on the motivation theory and Elaboration Likelihood Model (ELM), we integrated text mining and a negative binomial regression model to explore how the central route (i.e., quality of health information) and peripheral route (the efforts of responders) affect the helpfulness votes of health information in OMHCs. It is expected that our findings will enrich the existing theoretical research on information helpfulness, provide insight into the information evaluation mechanism of information searchers, and provide feasible suggestions for the sustainable development of the OMHCs.

## Theoretical background and hypotheses development

### Theoretical background

#### Motivation theory

Motivation theory holds that individual behaviors are normally driven by intrinsic and extrinsic motivation (Michaelson, 2005). Intrinsic motivation comes from the innate psychological need of individuals (e.g., altruism, curiosity, and avoiding harm), which is not affected by external incentive factors, while extrinsic motivation is generated by external incentives, such as the pursuit of rewards or recognition. For online community, motivation theory has been adopted to explain the individual behavior such as online contribution (Wang and Fesenmaier, 2003), evaluating online content (Zhang et al., 2015), knowledge sharing (Mojdeh et al., 2018), information exchange (Lee and Suzuki, 2020), sustained participation (Wu and Gong, 2021), and so on. Intrinsic motivation, which contains enjoyment in helping (Chang Y. et al., 2020), hedonic (Lai and Chen, 2014), self-efficacy (Wang and Lai, 2006) etc., plays a key role in exploring the influencing factors of individual behavior in online community. In contrast, reputation (Wu and Gong, 2021), reciprocity (Wang and Fesenmaier, 2004), social need (Chen et al., 2013), rewards (Maharani and Hendriyani, 2017) and so on, as extrinsic motivation, is equally important.

Online health community, a special virtual community which shared health knowledge in professional (Liang et al., 2011), has been widely studied by scholars and the effect of motivation mechanisms has an impact on it (Ba and Wang, 2013).As the related research further develops in OHC, motivation theory has been widely employed to explain individual knowledge sharing behavior in OHCs (Deng et al., 2019; Zhou et al., 2019). With respect to intrinsic motivation in knowledge sharing behavior, altruism, which means that people help others without any rewards just due to enjoyment, has no obvious main effect on it (Park and Gabbard, 2018) while the other scholar holds that altruism has a positive impact on it (Zhang et al., 2017b). Knowledge self-efficacy fully mediates the effects of social capital on knowledge sharing (Rains et al., 2016; Zhang et al., 2017a).

As for extrinsic motivation, reciprocity will positively affect knowledge sharing intention (Zhang et al., 2017b), requirement that users want to get a better understanding of current domain knowledge will influence the knowledge sharing (Hara and Foon Hew, 2007), reputation will have a positive impact on knowledge sharing behavior (Yan et al., 2016), compared with normal users, the health professionals will be more influential by reputation (Zhang et al., 2017b). In online mental health community, users prefer to seeking useful information from psychological counselor with higher reputation (Zhou et al., 2018). Reputation, as an external motivation, has been widely studied by scholars in the fields of health knowledge sharing, physicians’ group joining behavior and so on (Wu and Lu, 2016; Qiao et al., 2021). In this current study, we employ reputation as an external motivational factor to study information usefulness.

#### Elaboration likelihood model

Motivation plays a key role in the context of persuasion, and if participants are motivated by a certain element, the elaboration likelihood will be increased (Petty and Cacioppo, 1986; Yang et al., 2021). The ELM was proposed by Petty and Cacioppo in the 1980s (Petty et al., 1981). It provides a conceptual framework for understanding the basic yet potentially contradictory factor of effectiveness of communication (Petty and Cacioppo, 1986). The theory of ELM states that persuasion can occur *via* two pathways, namely the central route and the peripheral route. The former refers to the process by which users change their attitude or put forward a certain point of view by consciously refining the obtained core arguments. On the contrary, the peripheral route emerges when one has poor information-processing ability and relies primarily on external environmental factors to make a certain judgment (Luo et al., 2014). ELM holds that the dominance of either route depends on the level of users’ elaboration likelihood of the acquired information, and established information that will affect the change of attitude after the fine machining process (Pierro et al., 2004). ELM has been widely applied to a diverse set of research topics, such as online information dissemination (Bi et al., 2017), consumer behavior formulation (Chang H. H. et al., 2020; Bao and Wang, 2021), online health community (Li et al., 2021; Zhu et al., 2021) and so on.

Because of the professional information in OMHCs, users have different ability to discriminate specialized knowledge. Since users can judge the quality of information by processing information and measuring it from other perspectives, therefore, we take information quality as central route in this study, and take responders’ efforts as peripheral route. When the user’s information judgment ability is poor, he will make the decision of information quality through the timeliness or the interactive feedback, so responder efforts can be taken into consideration as peripheral route.

### Hypotheses

As mentioned above, if the information receivers pay attention to identifying the core content that might change their attitude, the central route will be dominant; otherwise, due to the scarcity of health knowledge and other external influential factors, the peripheral route will play the most important role in information perception and processing. According to existing studies, the quality of the given information is one of the main standards of measurement in the central route (Meservy et al., 2014). Information quality can refer to length, readability, similarity (which means that the relevation between the response and questions) and so on (Jin et al., 2016). In cases where the source of information is not included in the information itself, the peripheral route is often easily taken by users (Petty et al., 1981). Following this theoretical logic, we define information quality as being key to the central route, and measure it by using the length of health information, readability of health information, and response consistency of the response. The peripheral route depends on the responder’s ability to persuade people who are lack of information-processing capability, and the timeliness of the response and interactive feedback will be used to measure it.

#### The effect of the central route: Information quality

Typically, in most online knowledge-sharing community, the longer the information, the richer is the content. Therefore, the length of the content may stimulate users to browse more carefully. Previous studies have found that a greater length of online comments distinctly influences consumers to carefully read specific comments, rather than focus solely on the summary data of comment stars (Chevalier and Mayzlin, 2006). In addition, Zhang et al. examined the helpfulness votes of Amazon commodity reviews and found that the length of comments has a significant impact on their persuasion ability, i.e., consumers may be more likely to recognize such comments and cast useful votes (Zhang et al., 2010). Many studies on transactional communities also show that the length of comments has a positive impact on perceived information helpfulness (Huang et al., 2015; Chen et al., 2021). For complex mental health information, longer content is more usually more systematic and standardized, which normally contains the responders’ detailed and rational analysis of mental status of the help-seekers, thus other visitors are more likely to cast a higher helpfulness vote after reading. We propose Hypothesis 1 (H1):

*H1*: The length of health information positively affects users’ information helpfulness votes in OMHCs.

The reading and adoption of information depends on the reader’s thought process and understanding. The ability to understand the language and the level of knowledge required to assimilate the information reflect the level of readability (Korfiatis et al., 2012; Liu and Park, 2015) have shown that the readability of online comments has a great impact on their perceived helpfulness, i.e., highly readable comments will make users feel that the information contained in such comments is more useful. When the comment contains information related to product attributes and requires readers to make a judgment on account of their understanding, its readability has a more apparent impact on helpfulness (Wang et al., 2019). Due to the high professionalism of health and medical-related content, readers are required to have a certain cognition of health literacy (Yan and Tan, 2014). It would be easier for readers to understand health information with high readability and consequently, make helpful evaluations. We propose Hypothesis2 (H2):

*H2*: The readability of health information positively affects users’ helpfulness votes for information shared in OMHCs.

This study uses response consistency to measure whether the responder writes the content in relation to the description of the question, reflecting the extent to which the responder meets the requirements of the original poster. The existing research on reviews left on e-commerce platforms shows that the greater the proportion of content related to product attributes, the more attention it receives; then, it would also obtain a larger number of useful votes, and acquire more valuable references. OMHCs tend to attract patients in need and provide mutual assistance. When a patient’s health information needs to get targeted feedback, such communities can help resolve the issue and assist the patient in carrying out self-health management. Therefore, the higher the accuracy of the response in the context of the question, the more likely it is to satisfy the poster’s demands and be acceptable to other users, and it may obtain more helpful votes. We then propose Hypothesis 3 (H3):

*H3*: The accuracy of the response in the context of the question positively impacts users’ votes on information helpfulness in OMHCs.

#### The effect of the peripheral route: Responders’ efforts

If a comment is published earlier, it obtains a first-mover advantage and has a much higher probability that it will be read by potential consumers than other comments (Mudambi and Schuff, 2010). In addition, users are more cautious about the helpfulness of recent commodity reviews because their authenticity is likely yet to be verified. The longer the comments stay in the community, the more users may pay attention to and evaluate them, and so the possibility of obtaining more useful evaluations is higher. In the context of OMHCs, many patients have an urgent need for health and medical information and expect to get responses from other users as quickly as possible. Therefore, the more time a response is, the greater would be its probability of receiving votes for usefulness. Based on the above statement, Hypothesis 4 (H4) is proposed:

*H4*: The timeliness of responses positively affects the helpfulness votes given by users’ in OMHCs.

Online interactions and discussions affect the quality of ideas based on the degree of facilitator interaction, which is also called feedback integration (Zhu et al., 2019). Relevant research on socialized Q&A communities reveals that the higher the frequency of interactions between questioners and responders, the greater would be the rate of questioners receiving high-quality feedback (Zhou et al., 2014). Textual feedback would be beneficial to the users’ subsequent knowledge contribution because it could affect their self-efficacy in an intangible way (Wang et al., 2022). As compared to e-commerce platforms, social media platforms are more interactive, and a stronger relationship can be established between the original poster and the responder. The credibility of the source of comments is higher in this scenario, and the publisher’s perceived helpfulness of the eWOM of online comments is stronger (Poston and Speier, 2005). In addition, from the perspective of conformity in social psychology, when other users in the community observe the increase in the length of feedback, they tend to perceive a higher degree of objectivity and quality of this information. In the interactive process of health communication, the poster communicates in the form of comments, which can help other knowledge seekers to understand the relevant health information more easily. Therefore, the higher the degree of interactive feedback, the more likely it is for the information to obtain helpfulness votes. Therefore, this paper proposes Hypothesis 5 (H5):

*H5*: Interactive feedback positively affects users’ helpfulness votes for information in OMHCs.

#### Moderating effect of online reputation

Online reputation is the overall evaluation of users by the community (Resnick et al., 2000), which is generally measured by eWOM, popularity of answers, professionalism and other indicators like competence and sociability (Banerjee et al., 2017). According to the existing research, the reputation of the reviewer has a “premium effect” (Shapiro, 1983; Cheema, 2008). Then the reputation in OHCs is often considered to be the evaluation of doctors by patients in the form of eWOM and user online comment information. In OMHCs, online reputation is obtained through community users’ continuous participation in high-quality knowledge sharing, which not only reflects their personal image and popularity, but also reflects the degree of their health literacy (Hsu and Lin, 2008).

Research have showed that reputation, as the extrinsic motivation of health knowledge sharing behavior, can significantly and positively affect doctors’ continuous contribution of health information (Meng et al., 2021). Users with high online reputation show more frequent knowledge sharing behavior in order to maintain their own reputation. Consequently, those with high online reputation and health information literacy are the primary sources of information and knowledge contribution in such OMHCs. Due to the professionalism of health and medical knowledge, users with high online reputation often become the people who respond most frequently to community inquirers. It is easier to gather the trust of other users in the content that they share. Therefore, although common users may fully accept information in cognition, but may not show positive and useful support for their efforts (such as rapid response, feedback and interaction).

Previous studies have proven that external motivation plays a negative regulatory role in the impact of internal motivation on information-sharing intentions (Zhao et al., 2016). As an expression of reputation, rank level of users can significantly motivate users’ intention to continuous knowledge sharing. For users with high reputation, continuous contribution of high-quality information is more likely to improve their online reputation. However, for one, users’ high reputation in OMHCs are also likely perceived as knowledgeable opinion leaders (professionals and experienced users) by pedestrian users. For another, health professionals or experienced users with a high reputation generally have pressure to keep their positive profiles and choose to share knowledge through answering normal users’ questions (Zhou et al., 2018), which is time-consuming and may reduce their extra efforts invested into daily continues communication. Their prompt and rapid response to questions were usually taken for granted by other common visitors, which might weaken their helpfulness perceptions. Based on the above point of view, Hypothesis 6a (H6a) and Hypothesis 6b (H6b) are proposed:

*H6a*: Online reputation of responders negatively moderates the relationship between the timeliness of responses and information helpfulness votes by users in OMHCs.

*H6b*: Online reputation of responders negatively moderates the relationship between interactive feedback and information helpfulness votes by users in OMHCs.

**Figure 1:**
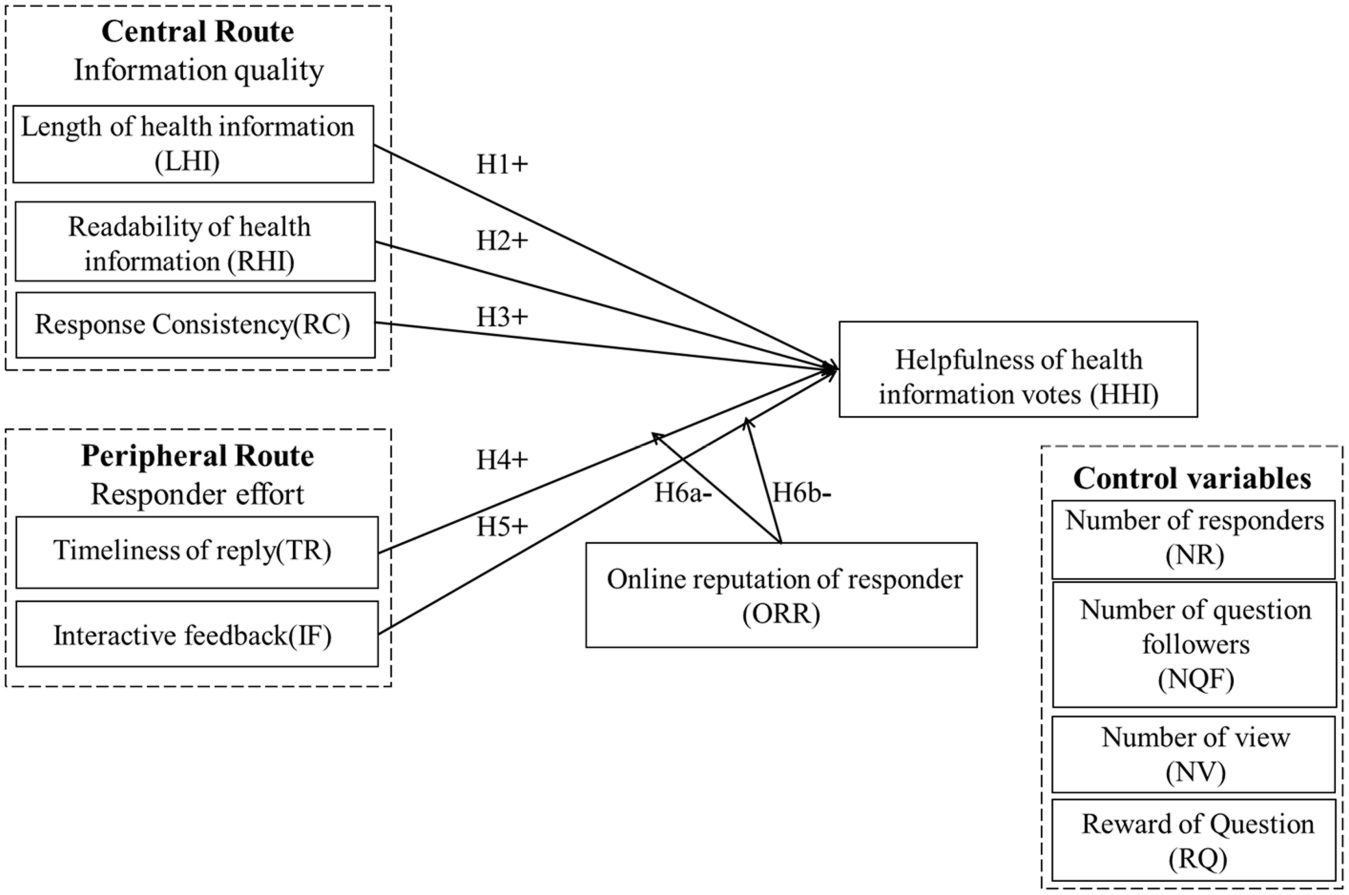
Research model.

The research model of this study is summarized in Figure.1.

## Materials and methods

### Data collection

The data used in this paper has been obtained from a well-known online mental health service website in China called *YiXinLi*.^1^ We crawled the data of the psychological question and answer forum through the Python crawler program. Before our data collection, our team members visited and interviewed with management team of counseling department in *YiXinLi* in Jun 2019 and was allowed to obtain some public Q&A data from this website.

**Figure 2:**
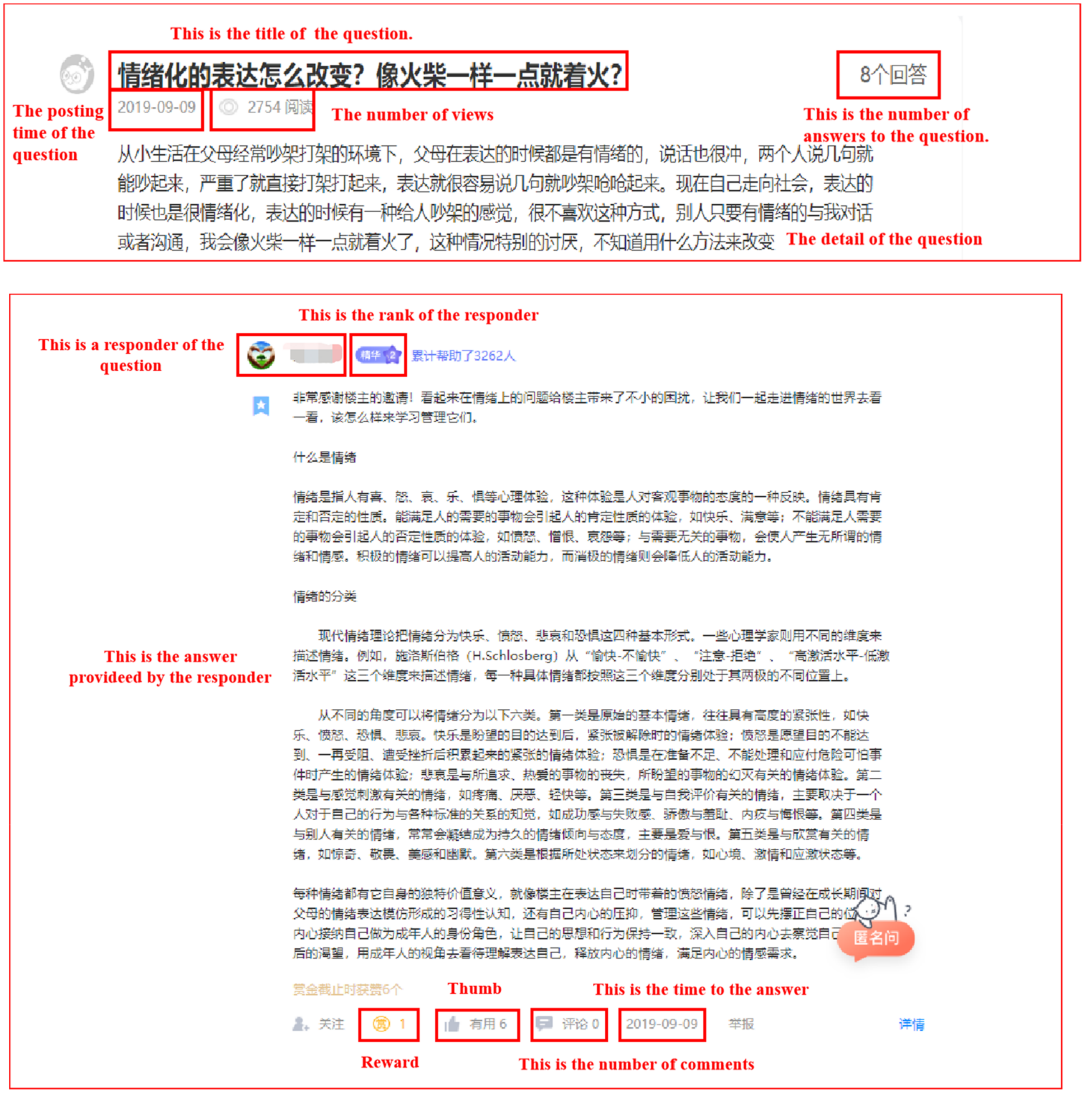
Example of question post and response post. Adapted with permission from https://www.xinli001.com/.

The data collection process includes three parts. Firstly, we chose the “recommended answer” of Q&A forum^2^, which will recommend and update latest answers every day. Secondly, we crawled the basic information of every question post including question title, question content, date of posting, the number of views, and so on. Thirdly, the detailed information of each answer post, such as responder’s nickname, response content, date of posting and the total number of rewards, thumb, comments were crawled as well. Through the above process, we collected the posting and response data of the Q&A community at *YiXinLi* in August, September and October, 2019, and thus integrated a 3-month cross-sectional dataset (a total of 5,252 question posts and 23,938 response posts in final dataset). Figure 2 shows one example of the question and answer post from *YiXinLi* Q&A forum.

A total of 11 variables were extracted from the abovementioned dataset. All variables and their descriptions are detailed in Table 1. The data is processed by Python-based text mining analysis and processing program, and the similarity between users’ questions and answers is characterized by cosine similarity.

**Table 1:** Variables and measurement.

Types	Variables	Measurement
Dependent variables	Helpfulness of health information votes (HHI)	The number of “thumb” received from other users in each response post, relative to health information shared by users
Independent variables	Length of health information (LHI)	The total number of words in the health information shared by responders
	Readability of health information (RHI)	Average length of each sentence (total length of text / punctuation number of text) in the content shared by the responders
	Response consistency (RC)	The text similarity between the content shared by the questioner and that shared by the responder
	Timeliness of reply (TR)	The inverse value of the interval between response time and question time
	Interactive feedback (IF)	Number of replies shared with other users in the process of message interaction
Moderator	Online reputation of responder (ORR)	The reputation level of the responder in the community
Control variables	Number of responders (NR)	The total number of responders to the same question post
	Number of question followers (NQF)	The number of collections of other users in the community
	Number of view (NV)	The total number of times the question was viewed by community users
	Reward of question (ROQ)	To measure whether a help-seeker offer money reward in question,1 for reward charge and 0 for free

### Measurement of variables

Based on the research of (Mudambi and Schuff, 2010) and other scholars on the quality of online comments, we measure information quality which is also considered as the central route shared by the responders from three perspectives: the length of health information content, the readability of such content and the degree of accuracy between the question and response. Among them, the length of content refers to the total textual volume of the shared content, which is a standard index used to measure quality in the field of information management (Khern-am-nuai et al., 2018). Since the length of health information only measures the volume of the content and is unable to describe its accuracy and relevance, we use the degree of response consistency, i.e., the degree of relevance between the information shared by users and the description provided in the question, as a supplement to the information quality. Specifically, we vectorize the text based on a Python program, which measures the similarity between the question text and the response text by using a cosine similarity algorithm (Lee et al., 2016). Generally speaking, readable information can improve the users’ understanding and reading experience. Therefore, we introduce the readability variable of information content (Korfiatis et al., 2012), and use the average number of characters contained in sentences in the responses to measure readability.

Responders’ effort is measured from the perspective of response timeliness and interactive feedback. The timeliness of the reply refers to the enthusiasm and promptness of the responder’s active reply to the user’s questions; interactive feedback refers to the behavior of the user who actively maintains a continuous interaction and channel of communication with the questioner. Users’ online reputation is measured by users’ reputation rating in the community. In addition, we consider the variables that may affect the evaluation of information helpfulness, such as the number of replies, the number of followers of the question, and the number of views of the question as the control variables (Lee et al., 2016). The specific methods employed for measuring the above variables are shown in Table 1.

## Results

The descriptive statistical results for the different variables are shown in Table 2 and it is produced by stata 15.0. It is obvious that the correlation coefficients of all variables are within a reasonable range, which avoids the impact of multi-collinearity of our research model in further analysis. In addition, expect the RHI and RC, other independent variables such as LHI, TR, IF positively correlate with HHI, which preliminarily verified our hypothesis. We thus test the relationship among the above variables by using econometric models.

**Table 2:** Results of descriptive statistics and covariance matrix.

Variables	HHI	NV	NQF	NR	ROQ	LHI	RHI	RC	TR	IF	ORR
HHI	1										
NV	0.223***	1									
NQF	0.185***	0.741***	1								
NR	0.192***	0.861***	0.561***	1							
ROQ	0.280***	0.284***	0.264***	0.283***	1						
LHI	0.585***	0.131***	0.119***	0.120***	0.221***	1					
RHI	−0.085***	0.026***	0.007	0.044***	−0.019***	−0.115***	1				
RC	0.006	−0.002	−0.004	−0.001	0.006	−0.001	0.002	1			
TR	0.036***	−0.184***	−0.165***	−0.160***	−0.146***	−0.026***	−0.006	0.007	1		
IF	0.095***	−0.017***	−0.027***	−0.015**	−0.005	0.053***	0.005	0.006	0.078***	1	
ORR	0.238***	−0.129***	−0.092***	−0.138***	0.020***	0.228***	−0.184***	0.006	0.143***	0.011*	1
Mean	2.499	408.660	3.366	9.592	0.658	345.442	0.084	0.194	28.760	0.347	4.979
S.D.	2.825	488.579	6.901	11.033	0.475	317.038	0.027	0.136	2.892	1.018	2.241

*p < 0.05, **p < 0.01, ***p < 0.001.

### Regression results

Since the dependent variable (helpfulness of health information) is a positive integer greater than 0, and the mean value of variables is inconsistent with the standard deviation, it is likely to produce over dispersion, therefore negative binomial regression model is more suitable for the data in this study. In addition, we use the variance inflation factor (VIF) value to test for multi-collinearity of the variables used in the regression model. It is found that the VIF value of all regression models is less than 5, indicating that multi-collinearity is relatively low.

The regression results, which can be seen in Table 3, are obtained by using the software Stata 15.0. Among these, model M1 tests the effect of the control variables. The model M2 tests the effects of LHI, RHI, RC, TR, and IF on the HHI. The empirical results show that the LHI has a significant positive impact on HHI votes (β = 0.002, *p* < 0.001), thus supporting H1. The RHI has a significant negative impact on the helpfulness of health information votes, and H2 is not supported (β = −2.345, *p* < 0.001). The impact of RC on HHI votes is also not significant, and H3 is also not verified.

**Table 3:** Result of negative binomial model regression and robustness test.

	DV: HHI (nberg)			DV: Reward-pay	DV: HHI (Zinb)	DV: log (HHI)
IV	M1	M2	M3	M4	M5	M6
NV	0.000*** (9.62)	0.000*** (9.49)	0.000*** (10.07)	0.000 (1.18)	0.000*** (13.08)	0.000*** (11.97)
NQF	0.008 (0.54)	−0.001 (−0.83)	0.000 (0.18)	0.004 (1.00)	−0.000 (−0.49)	−0.001 (−1.34)
NR	−0.001 (−1.20)	−0.003* (−2.47)	−0.000 (−0.74)	−0.009* (−2.51)	−0.004*** (−4.13)	−0.004*** (−4.62)
ROQ	0.701*** (43.43)	0.457*** (30.72)	0.452*** (30.90)	−0.114* (−2.36)	0.439*** (34.4)	0.394*** (34.95)
LHI		0.002*** (79.69)	0.002*** (75.33)	0.001*** (53.37)	0.001*** (52.71)	0.001*** (60.99)
RHI		−2.345*** (−8.19)	−0.804** (−2.87)	−2.930** (−2.66)	0.135 (0.49)	0.149 (1.62)
RC		0.048 (1.07)	0.041 (0.92)	−0.223 (−1.39.)	0.036 (0.95)	0.036 (1.00)
TR		0.035*** (15.19)	0.108*** (19.08)	0.100*** (5.04)	0.071*** (14.33)	0.067*** (16.15)
IF		0.068*** (11.79)	0.128*** (7.79)	0.197*** (5.67)	0.088*** (6.75)	0.085*** (6.45)
ORR			0.570*** (18.04)	0.538*** (5.38)	0.358*** (13.52)	0.342*** (14.02)
TR× ORR			−0.017*** (−15.65)	−0.017*** (−4.92)	−0.011*** (−11.89)	−0.010*** (−12.14)
IF ×ORR			−0.011*** (−3.65)	−0.016** (−2.94)	−0.007** (−2.84)	−0.006* (−2.48)
Constant	0.260*** (18.47)	−1.177*** (−15.91)	−3.7634*** (−22.56)	−5.797*** (−9.86)	−1.960*** (−13.34)	−2.123*** (−17.29)
lnalpha	−0.318	−0.922	−1.015	−25.890	−1.949	/
N	23,938	23,938	23,938	23,938	23,938	17,834
R2 (Pseudo R2)	0.0281	0.109	0.1185	0.113	/	0.325

*p < 0.05, **p < 0.01, ***p < 0.001; the parameters in brackets are t/z value.

In terms of the efforts of responders, the TR and IF have a significant positive effect on the HHI votes (β = 0.035, *p* < 0.001; β = 0.068, *p* < 0.001); therefore, both H4 and H5 are supported. Additionally, in the moderation effect test, both the interaction between RT and ORR, and the relationship between IF and ORR have a significant negative effect on HHI votes (β = −0.017, *p* < 0.001; β = −0.011, *p* < 0.001); thus, H6a and H6b are both supported.

## Robustness test

In order to further verify the robustness of the results, we conducted a series of robustness tests. Firstly, in previous research design, we used the number of “thumbs” objectively generated by the online health community to measure the helpfulness of health information votes. In the context of the knowledge economy, an increasing number of platforms are introducing reward-based mechanisms to influence user behavior. The platform studied in this paper allows users to pay for high-quality responses, and this willingness to pay reflects the high demand for information from users. Therefore, this paper further uses the number of times each reply is rewarded to measure the information helpfulness of user-generated content. As discussed, these regression results are shown in Table 3 (models M4). The significant test results of all assumptions are consistent with M1 to M3 above, indicating that the results are robust.

Secondly, since the real value of the dependent variable HHI has a large number of zeros, we use the zero-inflated negative binomial regression to control the potential deviation. The following result (M5) indicated that except the RHI is not significant compared with the original model, other effects of independent and moderated variables are consistent with the Model 3. In addition, given the abnormal distribution of the dependent variable HHI, a log transformation of the variable value and ordinary least squares (OLS) are used as the third robustness testing method. The significance of main variables in that results (M6) are consistent with the model 5 (M5), and we will make a summary and some possible explanations next.

The results are summarized as follows. Among these, all our hypotheses are supported apart from H2 and H3. For H2, low readability has a significant negative impact on information helpfulness. This result may be related to the specific situation of OMHCs. In addition to seeking emotional support in mental condition, inquirers may desire to obtain more professional information (such as psychological care or medical experience) in the process of searching for information. In such a scenario, if users feel that the content they read is relatively simple, understandable and familiar, they may reduce their votes on the helpfulness of the information content. For H3, the impact of question response consistency on the helpfulness of health information is not significant. The reason may be that this article is in an OMHC with mental health as the theme. The content of the response may not be limited to the specific questions of the inquirer, but might carry out in-depth analysis, put forward suggestions and express encouragement according to the content of the question. Therefore, the degree of accuracy of the responses to questions may not necessarily have a direct impact on information helpfulness.

## Conclusion and discussion

Based on the responses to questions in OMHCs, this paper discusses the impact of helpfulness of health information votes from an ELM perspective. Here, the information quality is regarded as the central route, and the responder efforts are the peripheral route. Based on the statement above, the empirical results show that: (1) in the central route, the length of health information has a significant positive impact on the users’ information helpfulness votes in OMHCs, and the readability of health information has a significant negative impact on the users’ information helpfulness votes, and (2) in the peripheral route, it can be seen that both the timeliness of users’ replies and interactive feedback have a significant positive impact on the users’ information helpfulness votes, while these positive effects are negatively moderated by the online reputation of responders.

### Theoretical contributions

Previous studies focused primarily on e-commerce platforms such as Amazon and Taobao, or general socialized Q&A communities such as *Zhihu* and *Baidu zhidao*, while a limited number of studies focus on the emerging nature of OMHCs. In this study, we discuss the helpfulness of information in combination with the inherent information quality and responders’ efforts from the perspective of motivation theory and employ ELM in OMHCs, and the research conclusion enriches the existing literature on users’ information behavior on other medical and health platforms.

Firstly, the current study identifies two persuasive routes underlying helpfulness of mental health votes: health information quality and responders’ effort. The empirical results show that although the information quality is the more obvious central evidence, the responders’ effort as distinct peripheral route have a more consistent and significant impact on information helpfulness voting. We expand the theory of ELM in OMHCs by figuring out that, for health-related information, it is normally difficult for common users to identify some high-quality information, but the usefulness of information can be determined by observing the efforts that information senders have invested. Additionally, compared with prior research on the helpfulness of online comments, this study came to some inconsistent conclusions. Many studies have shown that the readability and response consistency of information content have a positive effect on the helpfulness, but if the health information provided exceeds the scope of the problem description itself, the user helpfulness votes will decline.

Secondly, we have proven that the online reputation of help-provider in OMHCs, as an extrinsic motivation, could undermine the impact of prosocial behavior such as responder’s effort on information usefulness. Different from previous studies that have found the behavior characteristics of opinion leaders with a high reputation in the virtual community following the “Matthew Effect” (i.e., users with a high reputation may get more approval and recognition), our study found that in the context of OMHCs, the sense of trustworthiness developed by high-profile users would directly lead to the high information acceptance by other users without much evaluation of helpfulness. For users with a high reputation, their prosocial behaviors are more likely to be taken for granted by other users and received less helpful votes. On the contrary, the responders’ efforts of users with a low reputation are often more likely to elicit praise and recognition from other users in the community.

### Practical implications

On the one hand, from the perspective of users in OMHCs, publishing more comprehensive health information as replies may get more useful votes, but at the same time, attention must also be paid to the professionalism and preciseness of the health content. Although overly short and casual replies can result in a better reading experience, they may result in “already familiar,” “low credibility” and other cognitive biases on the part of other users. This would reduce the perceived helpfulness of the information. Due to lack of sufficient professional knowledge, common users may rely more on the speed of response and extra efforts of response to judge the usefulness of information. Therefore, prompt responses to the latest replies and continuous interactive feedback with the questioner can also improve their evaluation of helpfulness, which is beneficial for building their personal profiles. Particularly, for novice users who are unfamiliar to the platform and with low online reputation, their efforts to respond are more likely to gain the attention and recognition of others, which plays a positive role in building personal community reputation.

On the other hand, from the perspective of platform governance, there exist inconsistent incentives for users with different reputation levels in helpfulness voting. Generally, high-profile opinion leaders are the main source of community knowledge contribution, so their knowledge contribution may cause other users to “take it for granted,” and their efforts to reply may be recognized by users without making a much higher helpfulness evaluation. Therefore, in the IT affordances such as evaluation of information helpfulness, the platform should provide insight into the possible impact of the voting scoring mechanism. Given the negative impact of reputation on users’ efforts and helpfulness voting, the platform needs to establish a personalized growth system to meet the needs of users at different ranks. Different incentive mechanisms or information adoption mechanisms can be set according to the reputation level of users in the community, so as to fully reflect the information feedback of knowledge inquirers and encourage continuous responses from various users in the community.

## Limitations and future directions

Firstly, due to the availability of data, this study only analyzes a single online health community with 3 months data, we could not analyze the possible platform-related and time effect so that the generalization of the research conclusion may be limited. Further research can integrate and mine sample data from multiple health communities. Secondly, we do not distinguish the different types of inquiry in the question post which might affect different audiences. The follow-up research can identify and discuss different types of counseling by combining machine learning and other available methods. Thirdly, this paper uses cosine similarity based on semantic matching to measure the question response consistency variable, and there may be some biases in the results. Future research can extend this and use methods based on a combination of literal matching and semantic matching to obtain more accurate results.

## Data availability statement

Publicly available datasets were analyzed in this study. This data can be found at: https://www.xinli001.com.

## Author contributions

GW and XG: introduction, results, and draft writing. GW, XG, and SH: theoretical background and hypotheses development. GW and CG: method. GW: robustness test, conclusion, discussion, limitations, and future directions. CG: first draft review, project administration, and funding acquisition. GW, XG, CG, and SH: article modification. All authors contributed to the article and approved the submitted version.

## Funding

This research was funded by the Doctoral Research Startup Fund of Panzhihua University, and the Open Funding of Sichuan Industrial Development Research Center for Vanadium & Titanium Materials: the theme tourism research driven by the sustainable vanadium-titanium source in Panzhihua City under the Healthcare-based Industrial Model (035300593).

## Conflict of interest

The authors declare that the research was conducted in the absence of any commercial or financial relationships that could be construed as a potential conflict of interest.

## Publisher’s note

All claims expressed in this article are solely those of the authors and do not necessarily represent those of their affiliated organizations, or those of the publisher, the editors and the reviewers. Any product that may be evaluated in this article, or claim that may be made by its manufacturer, is not guaranteed or endorsed by the publisher.

## Footnotes

^1^ https://www.xinli001.com/

^2^ https://www.xinli001.com/qa?source=pc-home

## References

[ref1] Ba S. Wang L. (2013). Digital health communities: the effect of their motivation mechanisms. Decis. Support. Syst. 55, 941–947. doi: 10.1016/j.dss.2013.01.003

[ref2] Banerjee S. Bhattacharyya S. Bose I. (2017). Whose online reviews to trust? Understanding reviewer trustworthiness and its impact on business. Decis. Support. Syst. 96, 17–26. doi: 10.1016/j.dss.2017.01.006

[ref3] Bao Z. Wang D. (2021). Examining consumer participation on brand microblogs in China: perspectives from elaboration likelihood model, commitment-trust theory and social presence. J. Res. Interact. Mark. 15, 10–29. doi: 10.1108/JRIM-02-2019-0027

[ref4] Bi S. Liu Z. Usman K. (2017). The influence of online information on investing decisions of reward-based crowdfunding. J. Bus. Res. 71, 10–18. doi: 10.1016/j.jbusres.2016.10.001

[ref5] Chang Y. Hou R.-J. Wang K. Cui A. P. Zhang C.-B. (2020). Effects of intrinsic and extrinsic motivation on social loafing in online travel communities. Comput. Hum. Behav. 109:106360. doi: 10.1016/j.chb.2020.106360

[ref6] Chang H. H. Lu Y.-Y. Lin S. C. (2020). An elaboration likelihood model of consumer respond action to facebook second-hand marketplace: impulsiveness as a moderator. Inf. Manag. 57:103171. doi: 10.1016/j.im.2019.103171

[ref7] Cheema A. (2008). Surcharges and seller reputation. J. Consum. Res. 35, 167–177. doi: 10.1086/529532

[ref8] Chen L. Baird A. Straub D. (2019). Why do participants continue to contribute? Evaluation of usefulness voting and commenting motivational affordances within an online knowledge community. Decis. Support. Syst. 118, 21–32. doi: 10.1016/j.dss.2018.12.008

[ref9] Chen Y. Dai R. Wang L. Yang S. Li Y. Wei J. (2021). Exploring donor’s intention in charitable crowdfunding: intrinsic and extrinsic motivations. Ind. Manag. Data Syst. 121, 1664–1683. doi: 10.1108/IMDS-11-2020-0631

[ref10] Chen G. L. Yang S. C. Tang S. M. (2013). Sense of virtual community and knowledge contribution in a P 3 virtual community. Internet Res. 23, 4–26. doi: 10.1108/10662241311295755

[ref11] Chevalier J. A. Mayzlin D. (2006). The effect of word of mouth on sales: online book reviews. J. Mark. Res. 43, 345–354. doi: 10.1509/jmkr.43.3.345

[ref12] Deng Z. Hong Z. Zhang W. Evans R. Chen Y. (2019). The effect of online effort and reputation of physicians on patients’ choice: 3-wave data analysis of China’s good doctor website. J. Med. Internet Res. 21:e10170. doi: 10.2196/10170, PMID: 30848726 PMC6429049

[ref13] Diviani N. van den Putte B. Giani S. van Weert J. C. (2015). Low health literacy and evaluation of online health information: a systematic review of the literature. J. Med. Internet Res. 17:e112. doi: 10.2196/jmir.4018, PMID: 25953147 PMC4468598

[ref14] Guo S. Guo X. Fang Y. Vogel D. (2017). How doctors gain social and economic returns in online health-care communities: a professional capital perspective. J. Manag. Inf. Syst. 34, 487–519. doi: 10.1080/07421222.2017.1334480

[ref15] Hara N. Foon Hew K. (2007). Knowledge-sharing in an online community of health-care professionals. Inf. Technol. People 20, 235–261. doi: 10.1108/09593840710822859

[ref16] Hernández-Ortega B. (2018). Don’t believe strangers: online consumer reviews and the role of social psychological distance. Inf. Manag. 55, 31–50. doi: 10.1016/j.im.2017.03.007

[ref17] Hsu C.-L. Lin J. C.-C. (2008). Acceptance of blog usage: the roles of technology acceptance, social influence and knowledge sharing motivation. Inf. Manag. 45, 65–74. doi: 10.1016/j.im.2007.11.001

[ref18] Huang A. H. Chen K. Yen D. C. Tran T. P. (2015). A study of factors that contribute to online review helpfulness. Comput. Hum. Behav. 48, 17–27. doi: 10.1016/j.chb.2015.01.010

[ref19] Jiang Z. Benbasat I. (2004). Virtual product experience: effects of visual and functional control of products on perceived diagnosticity and flow in electronic shopping. J. Manag. Inf. Syst. 21, 111–147. doi: 10.1080/07421222.2004.11045817

[ref20] Jin J. Yan X. Li Y. Li Y. (2016). How users adopt healthcare information: an empirical study of an online Q&A community. Int. J. Med. Inform. 86, 91–103. doi: 10.1016/j.ijmedinf.2015.11.002, PMID: 26616406

[ref21] John B. M. Chua A. Y. K. Goh D. H. L. (2011). What makes a high-quality user-generated answer? IEEE Internet Comput. 15, 66–71. doi: 10.1109/MIC.2011.23

[ref23] Khern-am-nuai W. Kannan K. Ghasemkhani H. (2018). Extrinsic versus intrinsic rewards for contributing reviews in an online platform. Inf. Syst. Res. 29, 871–892. doi: 10.1287/isre.2017.0750

[ref24] Korfiatis N. García-Bariocanal E. Sánchez-Alonso S. (2012). Evaluating content quality and helpfulness of online product reviews: the interplay of review helpfulness vs. review content. Electron. Commer. Res. Appl. 11, 205–217. doi: 10.1016/j.elerap.2011.10.003

[ref25] Lai H.-M. Chen T. T. (2014). Knowledge sharing in interest online communities: a comparison of posters and lurkers. Comput. Hum. Behav. 35, 295–306. doi: 10.1016/j.chb.2014.02.004

[ref26] Lee G. M. Qiu L. Whinston A. B. (2016). A friend like me: modeling network formation in a location-based social network. J. Manag. Inf. Syst. 33, 1008–1033. doi: 10.1080/07421222.2016.1267523

[ref27] Lee G. Suzuki A. (2020). Motivation for information exchange in a virtual community of practice: evidence from a Facebook group for shrimp farmers. World Dev. 125:104698. doi: 10.1016/j.worlddev.2019.104698

[ref28] Li C.-R. Zhang E. Han J.-T. (2021). Adoption of online follow-up service by patients: an empirical study based on the elaboration likelihood model. Comput. Hum. Behav. 114:106581. doi: 10.1016/j.chb.2020.106581

[ref29] Liang H. Xue Y. Chase S. K. (2011). Online health information seeking by people with physical disabilities due to neurological conditions. Int. J. Med. Inform. 80, 745–753. doi: 10.1016/j.ijmedinf.2011.08.003, PMID: 21917511

[ref30] Liu Z. Park S. (2015). What makes a useful online review? Implication for travel product websites. Tour. Manag. 47, 140–151. doi: 10.1016/j.tourman.2014.09.020

[ref31] Liu S. Y. Zhang L. Xiang C. Liu Y.-T. Zhongchun H. Zhang S. B. (2020). Online mental health services in China during the COVID-19 outbreak. Lancet Psychiatry 7, e17–e18. doi: 10.1016/s2215-0366(20)30077-8, PMID: 32085841 PMC7129099

[ref32] Luo C. Wu J. Shi Y. Xu Y. (2014). The effects of individualism-collectivism cultural orientation on eWOM information. Int. J. Inf. Manag. 34, 446–456. doi: 10.1016/j.ijinfomgt.2014.04.001

[ref33] Maharani R. S. Hendriyani M. (2017). “The influence of motivation and social capital to knowledge sharing in online communities: study on female daily online community.” in Paper Presented at 1st Indonesia International Graduate Conference on Communication (IGCC) 2017.

[ref34] Meng F. Zhang X. Liu L. Ren C. (2021). Converting readers to patients? From free to paid knowledge-sharing in online health communities. Inf. Process. Manag. 58:102490. doi: 10.1016/j.ipm.2021.102490

[ref35] Meservy T. O. Jensen M. L. Fadel K. J. (2014). Evaluation of competing candidate solutions in electronic networks of practice. Inf. Syst. Res. 25, 15–34. doi: 10.1287/isre.2013.0502

[ref36] Michaelson C. (2005). Meaningful motivation for work motivation theory. Acad. Manag. Rev. 30, 235–238. doi: 10.5465/amr.2005.16387881

[ref37] Mojdeh S. Head M. El Shamy N. (2018). Knowledge sharing in social networking sites: how context impacts individuals’ social and intrinsic motivation to contribute in online communities. AIS Trans. Hum. Comp. Inter. 10, 82–104. doi: 10.17705/1thci.00105

[ref38] Mudambi S. M. Schuff D. (2010). Research note: what makes a helpful online review? A study of customer reviews on Amazon.com. MIS Q. 34, 185–200. doi: 10.2307/20721420

[ref39] Park J. Gabbard J. L. (2018). Factors that affect scientists’ knowledge sharing behavior in health and life sciences research communities: differences between explicit and implicit knowledge. Comput. Hum. Behav. 78, 326–335. doi: 10.1016/j.chb.2017.09.017

[ref40] Petty R. E. Cacioppo J. T. (1986). “The elaboration likelihood model of persuasion,” in Communication and Persuasion: Central and Peripheral Routes to Attitude Change. eds. Petty R. E. Cacioppo J. T. (New York, NY: Springer New York), 1–24.

[ref41] Petty R. E. Cacioppo J. T. Goldman R. (1981). Personal involvement as a determinant of argument-based persuasion. J. Pers. Soc. Psychol. 41, 847–855. doi: 10.1037/0022-3514.41.5.847

[ref42] Pierro A. Mannetti L. Kruglanski A. W. Sleeth-Keppler D. (2004). Relevance override: on the reduced impact of “cues” under high-motivation conditions of persuasion studies. J. Pers. Soc. Psychol. 86, 251–264. doi: 10.1037/0022-3514.86.2.251, PMID: 14769082

[ref43] Poston R. S. Speier C. (2005). Effective use of knowledge management systems: a process model of content ratings and credibility indicators. MIS Q. 29, 221–244. doi: 10.2307/25148678

[ref44] Qazi A. Shah Syed K. B. Raj R. G. Cambria E. Tahir M. Alghazzawi D. (2016). A concept-level approach to the analysis of online review helpfulness. Comput. Hum. Behav. 58, 75–81. doi: 10.1016/j.chb.2015.12.028

[ref45] Qianqian Ben L. Xiaoxiao L. Xitong G. (2020). The effects of participating in a physician-driven online health community in managing chronic disease: evidence from two natural experiments. MIS Q. 44, 391–419. doi: 10.25300/MISQ/2020/15102

[ref46] Qiao W. Yan Z. Wang X. (2021). Join or not: the impact of physicians’ group joining behavior on their online demand and reputation in online health communities. Inf. Process. Manag. 58:102634. doi: 10.1016/j.ipm.2021.102634

[ref47] Qigeng L. Xiaohong Z. Yaoyu H. (2017). An empirical study of influencing factors of perceived usefulness of online review. Inform. Stud. Theory Appl. 40, 122–125. doi: 10.16353/j.cnki.1000-7490.2017.08.022

[ref48] Racherla P. Friske W. (2012). Perceived “usefulness” of online consumer reviews: an exploratory investigation across three services categories. Electron. Commer. Res. Appl. 11, 548–559. doi: 10.1016/j.elerap.2012.06.003

[ref49] Rains S. A. Brunner S. R. Akers C. Pavlich C. A. Goktas S. (2016). Computer-mediated communication (CMC) and social support: testing the effects of using CMC on support outcomes. J. Soc. Pers. Relat. 34, 1186–1205. doi: 10.1177/0265407516670533

[ref50] Resnick P. Zeckhauser R. Friedman E. Kuwabara K. O. (2000). Reputation systems. Commun. ACM 43, 45–48. doi: 10.1145/355112.355122

[ref51] Salloum S. A. Al-Emran M. Shaalan K. (2018). “The impact of knowledge sharing on information systems: a review,” in Knowledge Management in Organizations. eds. Uden L. Hadzima B. (Ting: Springer International Publishing), 94–106.

[ref52] Shapiro C. (1983). Premiums for high quality products as returns to reputations. Q. J. Econ. 98, 659–679. doi: 10.2307/1881782

[ref53] Wang Y. Fesenmaier D. R. (2003). Assessing motivation of contribution in online communities: an empirical investigation of an online travel community. Electron. Mark. 13, 33–45. doi: 10.1080/1019678032000052934

[ref54] Wang Y. Fesenmaier D. R. (2004). Towards understanding members’ general participation in and active contribution to an online travel community. Tour. Manag. 25, 709–722. doi: 10.1016/j.tourman.2003.09.011

[ref55] Wang C.-C. Lai C.-Y. (2006). “Knowledge contribution in the online virtual community: capability and motivation,” in Knowledge Science, Engineering and Management. eds. Lang J. Lin F. Wang J. (Heidelberg: Springer), 442–453.

[ref56] Wang N. Liu Y. Xiao S. (2022). Which feedback matters? The role of expressions and valence in continuous high-quality knowledge contribution in the online Q&A community. Decis. Support. Syst. 156:113750. doi: 10.1016/j.dss.2022.113750

[ref57] Wang Y. Wang J. Yao T. (2019). What makes a helpful online review? A meta-analysis of review characteristics. Electron. Commer. Res. 19, 257–284. doi: 10.1007/s10660-018-9310-2

[ref58] Wu W. Gong X. (2021). Motivation and sustained participation in the online crowdsourcing community: the moderating role of community commitment. Internet Res. 31, 287–314. doi: 10.1108/INTR-01-2020-0008

[ref59] Wu H. Lu N. (2016). How your colleagues’ reputation impact your patients’ odds of posting experiences: evidence from an online health community. Electron. Commer. Res. Appl. 16, 7–17. doi: 10.1016/j.elerap.2016.01.002

[ref60] Yan Z. Kuang L. Qiu L. (forthcoming). Prosocial behaviors and economic performance: evidence from an online mental healthcare (November 25, 2019). Production and Operations Management. Available at SSRN: https://ssrn.com/abstract=3493138

[ref61] Yan L. Tan Y. (2014). Feeling blue? Go online: an empirical study of social support among patients. Inf. Syst. Res. 25, 690–709. doi: 10.1287/isre.2014.0538

[ref62] Yan Z. Wang T. Chen Y. Zhang H. (2016). Knowledge sharing in online health communities: a social exchange theory perspective. Inf. Manag. 53, 643–653. doi: 10.1016/j.im.2016.02.001

[ref63] Yang S. Zhou C. Chen Y. (2021). Do topic consistency and linguistic style similarity affect online review helpfulness? An elaboration likelihood model perspective. Inf. Process. Manag. 58:102521. doi: 10.1016/j.ipm.2021.102521

[ref630] Zhang K. Z. Lee M. K. Zhao S. J. (2010). “Understanding the informational social influence of online review platforms.” in ICIS 2010 Proceedings. 71.

[ref64] Zhang X. Liu S. Chen X. Gong Y. (2017a). Social capital, motivations, and knowledge sharing intention in health Q&A communities. Manag. Decis. 55, 1536–1557. doi: 10.1108/MD-10-2016-0739

[ref65] Zhang X. Liu S. Deng Z. Chen X. (2017b). Knowledge sharing motivations in online health communities: a comparative study of health professionals and normal users. Comput. Hum. Behav. 75, 797–810. doi: 10.1016/j.chb.2017.06.028

[ref66] Zhang T. Wang W. Y. C. Lin Y. C. Tai L.-H. (2015). Understanding user motivation for evaluating online content: a self-determination theory perspective. Behav. Inform. Technol. 34, 479–491. doi: 10.1080/0144929X.2014.964319

[ref67] Zhao L. Detlor B. Connelly C. E. (2016). Sharing knowledge in social Q & a sites: the unintended consequences of extrinsic motivation. J. Manag. Inf. Syst. 33, 70–100. doi: 10.1080/07421222.2016.1172459

[ref68] Zhou J. (2018). Factors influencing people’s personal information disclosure behaviors in online health communities: a pilot study. Asia Pac. J. Public Health 30, 286–295. doi: 10.1177/1010539518754390, PMID: 29405740

[ref69] Zhou J. Amo L. Ye C. Kai S. (2018). “Using reputation to predict online psychological counselor appointment.” in IGMIS-CPR ‘18: 2018 Computers and People Research Conference. 107–108.

[ref710] Zhou J. Kishore R. Amo L. Ye C. (forthcoming). Description and demonstration signals as complements and substitutes in an online market for mental healthcare MIS Q. doi: 10.25300/MISQ/2022/16122

[ref70] Zhou J. Zuo M. Ye C. (2019). Understanding the factors influencing health professionals’ online voluntary behaviors: evidence from YiXinLi, a Chinese online health community for mental health. Int. J. Med. Inform. 130:103939. doi: 10.1016/j.ijmedinf.2019.07.018, PMID: 31434043

[ref71] Zhou J. Zuo M. Yu Y. Chai W. (2014). How fundamental and supplemental interactions affect users’ knowledge sharing in virtual communities? A Soc. Cogn. Persp Int. Res. 24, 566–586. doi: 10.1108/IntR-07-2013-0143

[ref72] Zhu H. Kock A. Wentker M. Leker J. (2019). How does online interaction affect idea quality? The effect of feedback in firm-internal idea competitions. J. Prod. Innov. Manag. 36, 24–40. doi: 10.1111/jpim.12442

[ref73] Zhu M. Wu C. Huang S. Zheng K. Young S. D. Yan X. (2021). Privacy paradox in mHealth applications: an integrated elaboration likelihood model incorporating privacy calculus and privacy fatigue. Telematics Inform. 61:101601. doi: 10.1016/j.tele.2021.101601

